# 

**Published:** 1874-12-15

**Authors:** 


### CONTENTS:

*ORIGINAL COMMUNICATIONS:*

Clinical Cases in the Mercy Hospital, involving Obscure Nervous and Cerebral Symptoms. By N. S. Davis, M. D. ........ 591

*CLINICAL REPORTS:*

Clinical Lecture in the Ophthalmic Department of Cook Co. Hospital. By F. C. Hotz, M. D. 595

*TRANSLATIONS:*

Progress of Medical Science in Germany. By E. J. Doering, M.D ........ 597

The Application of Massage (*Kneading*) and Steam, in Abscess of the Cornea ........ 601

Gleanings from the French ........ 603

*EDITORIAL DEPARTMENT* ........ 605

*SOCIETY REPORTS:*

The American Public Health Association—Second Annual Session ........ 606

*GLEANINGS FROM OUR EXCHANGES:*

The Treatment of Pertussis by Inhalation ........ 617

Klein on the Anatomical Changes in Typhoid Fever; Scarlatinal Waves ........ 618

*BOOK REVIEWS:* ........ 620

